# Twin SVM-Based Classification of Alzheimer's Disease Using Complex Dual-Tree Wavelet Principal Coefficients and LDA

**DOI:** 10.1155/2017/8750506

**Published:** 2017-08-16

**Authors:** Saruar Alam, Goo-Rak Kwon, Ji-In Kim, Chun-Su Park

**Affiliations:** ^1^Department of Information and Communication Engineering, Chosun University, 375 Seosuk-Dong, Dong-Gu, Gwangju 501-759, Republic of Korea; ^2^Department of Computer Education, Sungkyunkwan University, 05006 209 Neungjong-ro, Gwangjin-gu, Seoul, Republic of Korea

## Abstract

Alzheimer's disease (AD) is a leading cause of dementia, which causes serious health and socioeconomic problems. A progressive neurodegenerative disorder, Alzheimer's causes the structural change in the brain, thereby affecting behavior, cognition, emotions, and memory. Numerous multivariate analysis algorithms have been used for classifying AD, distinguishing it from healthy controls (HC). Efficient early classification of AD and mild cognitive impairment (MCI) from HC is imperative as early preventive care could help to mitigate risk factors. Magnetic resonance imaging (MRI), a noninvasive biomarker, displays morphometric differences and cerebral structural changes. A novel approach for distinguishing AD from HC using dual-tree complex wavelet transforms (DTCWT), principal coefficients from the transaxial slices of MRI images, linear discriminant analysis, and twin support vector machine is proposed here. The prediction accuracy of the proposed method yielded up to 92.65 ± 1.18 over the Alzheimer's Disease Neuroimaging Initiative (ADNI) dataset, with a specificity of 92.19 ± 1.56 and sensitivity of 93.11 ± 1.29, and 96.68 ± 1.44 over the Open Access Series of Imaging Studies (OASIS) dataset, with a sensitivity of 97.72 ± 2.34 and specificity of 95.61 ± 1.67. The accuracy, sensitivity, and specificity achieved using the proposed method are comparable or superior to those obtained by various conventional AD prediction methods.

## 1. Introduction

Alzheimer's disease (AD) is the most familiar cause of dementia, with patients comprising 50%–80% of all dementia sufferers. The disease affects memory, cognition, and behavior. As AD is a neurodegenerative condition, several types of atrophy occur in the hippocampus and other areas of the brain. Despite being the 6th leading cause of death in the USA, it is not a common disease. Currently, there is no cure; however, some preventive measures can be taken to mitigate risk factors and slow the degenerative process. An estimated $605 billion globally and $220 billion in USA is spent annually on diagnosing AD. Many people suffer from AD worldwide, and demands on researchers are growing rapidly. MRI is an effective medical image construction technique, as it has the proven potential to view structural changes in the human brain, internal organs, and other tissues.

MRI produces high-quality structural images, providing distinctive tissue information, which enhances both the accuracy of brain pathology diagnosis and quality of treatment. A key advantage of this technique is its noninvasiveness. Many studies have been conducted using multivariate analysis algorithms and structural/functional MRI to classify neurological diseases [1–3]. A primary focus of these studies was the large dimensionality of extracted features and the identification of disease signatures among them where the most discriminative information of the said diseases exists. Results showed significant cerebral structural changes in several brain ROIs, particularly in the hippocampus and entorhinal cortex [4]. Global and internal intensity-based features, [3, 5], as well as geometric- and surface-based features [6, 7], have been used in earlier studies for classifying disease. The authors presented an electroencephalogram (EEG) coherence study of Alzheimer's disease using a probabilistic neural network (PNN) and showed significant accuracy in distinguishing true AD from the control groups [8]. Chaplot et al. [9] stratified AD using discrete wavelet coefficients as a feature for training and testing Support Vector Machines (SVMs) and neural network classifiers. Extracting essential discriminatory features from MRI brain images is imperative for competent analysis of disease diagnosis. The preferred feature extraction methods, amongst those most frequently used, are independent component analysis [10], wavelet transform [11], and Fourier transform [12]. This study has been conducted using discrete wavelet features and the k-nearest neighbor algorithm (k-NN) [11] on an artificial neural network (ANN) [11, 13]. Zhang and Wang [14] ran AD prediction models using displacement field estimation between AD and healthy controls using an SVM, twin support vector machine (TWSVM), and generalized eigenvalue proximal SVM (GEPSVM) as classifiers. Tomar and Agarwal [15] reviewed several types of twin SVM algorithms, their optimization problems, and their applications.

The biomarkers used in our proposed method are MRI images from the Alzheimer's Disease Neuroimaging Initiative (ADNI) and Open Access Series of Imaging Studies (OASIS) datasets. Our primary reason for using DTCWT over DWT is its effective representation of singularities (curves and lines), even though DWT has the advantage of representing the functions in multiscale and compressed forms. In DTCWT, shifts in magnitude variance can be achieved to a higher degree [16]. In our proposed method, DTCWT coefficient-based AD classification has been proposed using principal component analysis and linear discriminant analysis of extracted coefficients; a TWSVM was utilized as a supervising technique. Classification performance is documented regarding accuracy, sensitivity, and specificity, after applying 10-fold cross validation and running the program 10–20 times. Our method produced superior results when compared with several conventional AD classification methods.

## 2. Material and Methods

A total of 172 subjects from the ADNI dataset were used—86 AD and 86 HC. In addition, we used 95 subjects from the OASIS dataset—44 HC and 51 subjects suffering from very mild to mild AD.

### 2.1. Overview of Experimental Data

Data used in the preparation of this article were obtained from the Alzheimer's Disease Neuroimaging Initiative (ADNI) database (http://adni.loni.usc.edu).

The ADNI was launched in 2003 as a public-private partnership led by Principal Investigator Michael W. Weiner, MD. The primary goal of the ADNI is to test whether serial MRI, positron emission tomography (PET), other biological markers, and clinical and neuropsychological assessment can be combined to measure the progression of MCI and early-onset Alzheimer's disease AD. For up-to-date information, visit www.adni-info.org. The demographic details of data used from the ADNI are shown in Table 1.

In addition, we utilized MRI images downloaded from the OASIS dataset. OASIS is a database designed to compile MRI datasets and make them freely accessible to the scientific community. OASIS compiles two types of data: cross-sectional MRI data and longitudinal MRI data. Our study utilized cross-sectional MRI data, as our aims are to develop an automatic system for detecting AD, for which longitudinal MRI data is not optimal.

The OASIS dataset consists of 416 subjects aged between 18 and 96 years. Our study included 51 AD patients (35 with CDR = 0.5 and 16 with CDR = 1) out of 100 having dementia and 44 HC out of 98 normal subjects.  Table 2 shows the demographic details of the subjects used in our study. Both men and women are included and all subjects are right handed. The scale of the CDR is listed in Table 3.

### 2.2. Proposed Approach

**Figure 1:**
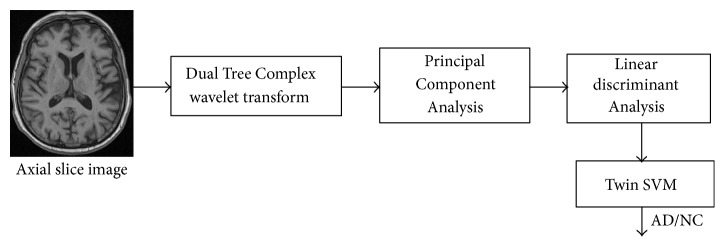
Flowchart of DTCWT-based classification performance of AD from HC.

The proposed approach is made up of 4 phases: preprocessing and slice extraction, feature extraction, projection of features into lower dimension, and efficient classification of the disease.  Figure 1 shows all phases in detail.

#### 2.2.1. Preprocessing and Slice Extraction

**Figure 2:**
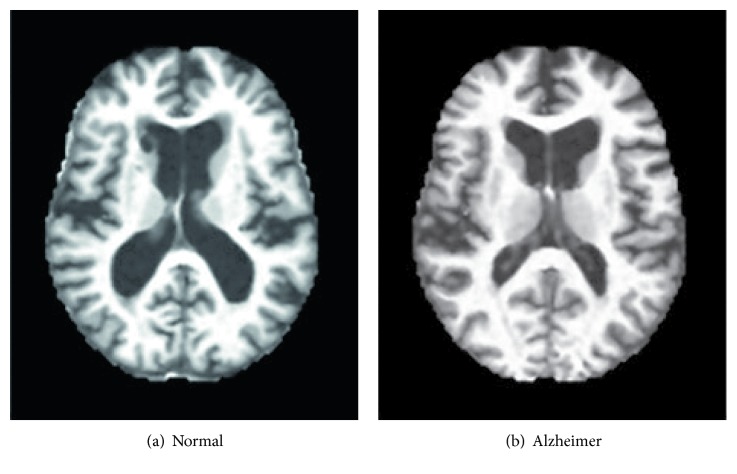
MR image slice sample (axial slice view after preprocessing).

All MRI images used for training and testing the TSVM of our proposed approach are viewed using the ONIS toolbox and exported as 2D MRI image slices. All images are in PNG format, and the dimensions of OASIS image slices are 176 × 208; the dimensions of the ADNI image slices are 256 × 166. The range of selection of those slices was performed manually from the tissue center for information clarity. The images are resized to 256 × 256 for further processing. A sample of a brain image slice is depicted in Figure 2. LibSVM toolbox was used for kernel SVM simulation in MATLAB.

#### 2.2.2. Dual-Tree Complex Wavelet Transform

**Figure 3:**
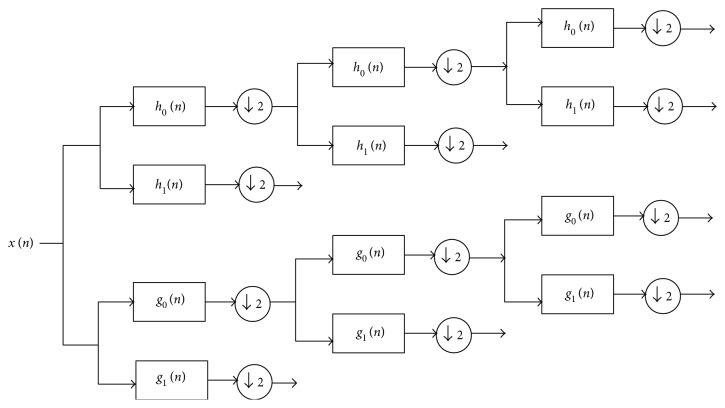
Block diagram for a 3-level DTCWT.

Wavelet transform (WT) is one of the most frequently used feature extraction techniques for MR images. For our proposed approach, we extract the DTCWT [16] coefficients from the input MRI images. The features of the 5th resolution scale were used as they produced higher classification performance when compared with other resolution levels. DTCWT has a multiresolution representation, as with CWT. For efficient disease classification, it is preferable to use a few intermediate scales of the extracted coefficients as input to a classifier, as the lowest resolution scales lose fine details and high-resolution scales contain mostly noise. Thus, we prefer to choose a few intermediate scales of DTCWT coefficients. These coefficients were sent as input for principal component analysis (PCA). CWT can be represented as complex-valued scaling functions and complex-valued wavelets. DTCWT engages two real DWTs, which provide the real and imaginary components of the wavelet transform, respectively. In addition, two filter bank types are set: analysis filter banks and synthesis filter banks. These filter banks are used for implementing DTCWT to ensure that overall transformation becomes almost analytic, as shown in Figure 3.

The DTCWT can be denoted in matrix form as

(1) $$\begin{array}{c} D=\left[D_{h}D_{g}\right], \end{array}$$

where *D*_*h*_ and *D*_*g*_ are rectangular matrices.

For the input image *x*, complex wavelet coefficients can be represented as

(2) $$\begin{array}{c} T_{h}+{jT}_{g}, \end{array}$$

where *T*_*h*_ = *D*_*h*_^∗^*x* is the real component and *T*_*g*_ = *D*_*g*_^∗^*x* is the imaginary part.

The DTCWT coefficients of input images are shift invariant; they do not change when an image is shifted in time or space. In addition, DTCWT employs segregation of 6 diverse directions (±15, ±30, and ±45) for 2D images and 28 different directions for 3D images, while conventional DWT only allows for isolation of horizontal and vertical directions. For each 2D slice subject image, we extracted 5-level DTCWT coefficients from one scale.

#### 2.2.3. Principal Component Analysis

**Figure 4:**
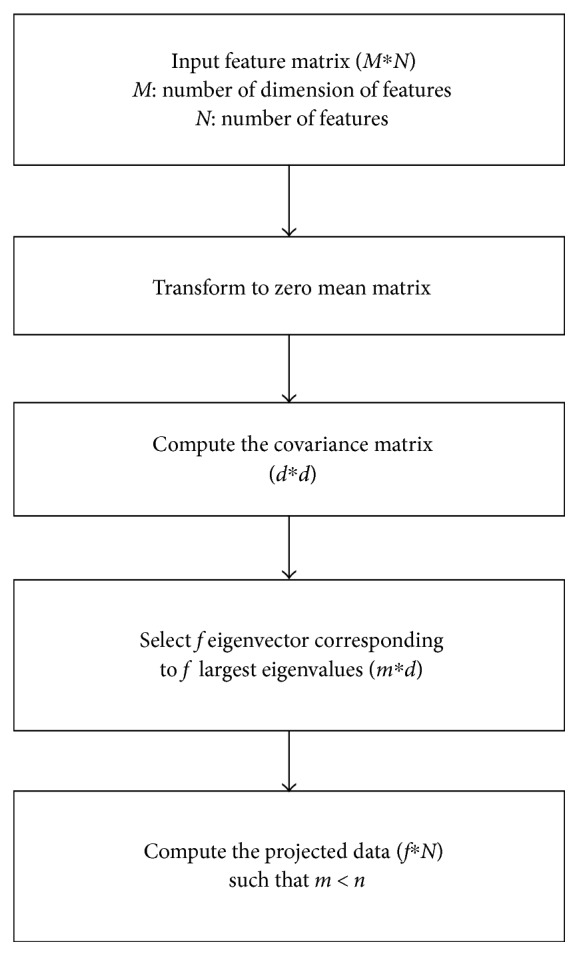
PCA implementation for feature reduction.

Principal component analysis (PCA) [17] is a dimensionality reduction technique that is applied to map features onto lower dimensional space. This data transformation may be linear or nonlinear. One of most frequently used linear transformation is PCA, which is an orthogonal transformation used to convert possibly correlated samples to linearly uncorrelated variables. The number of principal components is lower than or equal to the number of original variables. The PCA conversion process is shown in Figure 4.

The PCA is summarized as follows:

- Calculating the mean of the data and zero mean data
- Constructing the covariance matrix
- Acquiring the eigenvalue and the eigenvector
- Projecting the data matrix with eigenvectors corresponding to the highest to lowest eigenvalues.

#### 2.2.4. Linear Discriminant Analysis

A generalized Fisher linear discriminant [18] is used for the linear projection of features to separate two or more classes. To make effective and discriminative projected features, PCA coefficients can be projected on to a new LDA projection axis.

To find the class separation projection axis, it is necessary to determine between-class scatter and within-class variability.

The between class variable matrix can be denominated by sample variance as

(3) $$\begin{array}{c} S_{B}=\frac{1}{c}\overset{c}{\underset{j=1}{\sum }}\left(m_{j}-m\right){\left(m_{j}-m\right)}^{T}. \end{array}$$

Within class variance matrix can be expressed as

(4) $$\begin{array}{c} S_{w}=\overset{c}{\underset{j=1}{\sum }}\underset{z_{k}\in w_{i}}{\sum }\left(z_{k}-m_{i}\right){\left(z_{k}-m_{i}\right)}^{T}, \end{array}$$

where *z*_*k*_ is *k*th sample variable belonging to a class.

The generalized Rayleigh coefficient is

(5) $$\begin{array}{c} J\left(w\right)=\frac{W^{t}S_{B}W}{W^{t}S_{w}W}, \end{array}$$

where *W* is the matrix for LDA coefficients. This can be characterized using the generalized eigenvalue problem as

(6) $$\begin{array}{c} S_{B}W=\lambda S_{w}W, \end{array}$$

where *λ* is the eigenvalue.

If *S*_*w*_ is singular matrix, (6) can be simplified as

(7) $$\begin{array}{c} {S_{w}}^{-1}S_{B}W=\lambda W, \end{array}$$

where the eigenvectors of *S*_*w*_^−1^*S*_*B*_ will be *W*. The eigenvector matrix will be *W*_LDA_,

(8) $$\begin{array}{c} W_{LDA}=\left[W_{1}W_{2}W_{3}\cdots W_{k}\right],k\in Z. \end{array}$$

The PCA coefficients can be projected onto *l* lower dimensional LDA projection termed by eigenvectors corresponding nonzero higher energy eigenvalues,

(9) $$\begin{array}{c} {W_{LDA}}^{\prime }=\left[W_{1}W_{2}W_{3}\cdots W_{l}\right],l\in Z, \end{array}$$

where *l*  ≤  *k*.

The final feature matrix *F* is evaluated as

(10) $$\begin{array}{c} F={\left({W_{LDA}}^{\prime }\right)}^{T}\cdot \psi {\left(x\right)}_{pc}. \end{array}$$

#### 2.2.5. Twin Support Vector Machine

Jayadeva and Chandra [19] proposed a novel dual hyperplane-based variant twin SVM. The concepts of generalized eigenvalues proximal support vector machine (GEPSVM) are applied here, which require two nonparallel optimum hyperplanes for each class. There are two quadratic programming (QP) problems optimized as TSVM pairs, as in a typical SVM.

Mathematically, the TSVM primal problem can be optimized by solving the following two quadratic programming problems:

(11) $$\begin{array}{c} \underset{w_{1},b_{1},q}{\min}\frac{1}{2}{\left(X_{1}w_{1}+o_{1}b_{1}\right)}^{T}\left(X_{1}w_{1}+o_{1}b_{1}\right)+C_{1}{o_{2}}^{T}\xi_{1}\\ s.t.-\left(X_{2}w_{1}+o_{2}b_{1}\right)+\xi_{1}\geq o_{2},\xi_{1}\geq 0, \end{array}$$

(12) $$\begin{array}{c} \underset{w_{1},b_{1},q}{\min}\frac{1}{2}{\left(X_{2}w_{2}+o_{2}b_{2}\right)}^{T}\left(X_{2}w_{2}+o_{2}b_{2}\right)+C_{2}{o_{1}}^{T}\xi_{2}\\ s.t.-\left(X_{1}w_{2}+o_{1}b_{2}\right)+\xi_{2}\geq o_{1},\xi_{2}\geq 0. \end{array}$$

Here, *X*_*i*_ (*i* = 1, 2) are input features, *w*_*i*_ (*i* = 1, 2) are the normal hyperplane vectors, *b*_*i*_ (*i* = 1, 2) are bias terms, *C*_*i*_ (*i* = 1, 2) are the vectors of positive penalty parameters, *o*_*i*_ (*i* = 1, 2) are the suitable dimensional matrices of ones, and *ξ*_*i*_ (*i* = 1, 2) are the slack variables. Hence, the TSVM finds two hyperplanes, each of which is nearer to the data sample of one class than to that of another. Therefore, minimizing (11) and (12) will compel the hyperplanes to approximate the data of each class and enhance the classification rate. The optimization problem can be solved in the Lagrange duality principle [15].

## 3. Results and Discussions

### 3.1. Background

In this article, our proposed approach is presented using Fisher linear discriminant analysis of DTCWT principal components. The details of our proposed method are shown in Figure 1. The advantage of WT over FT is its multiple-scaled representations and frequency components with spatial domain information. Fourier coefficients only produce image frequency information, whereas wavelets contain powerful observations of the spatial and frequency domain in a multiscaled format. In addition, wavelet representation is spatially localized; Fourier functions are not spatially localized as they consist only of image frequency components. MRI images can be represented and processed at numerous resolutions and can therefore be used as an incisive framework for processing multiresolution images. Finally, DWT coefficients can be extracted by using arrays of low and high pass filter banks.

However, there are multiple drawbacks to conventional wavelet transform. These include drift in wavelet coefficient oscillation towards positive and negative around singularities, shift variance of signal (which may cause oscillation of wavelet coefficient samples around singularities), substantial aliasing of amply spaced wavelet coefficient patterns, and lack of directional selectivity perturbs to process and model geometric image features (such as edges and ridges). In these cases, flaws regarding conventional DWT are not experienced by Fourier transform. Inspired by Fourier transform, our improved DTCWT is used to overcome these drawbacks. Previous studies have shown that DTCWT feature-based AD disease detection performs better than typical DWT-based feature extraction [20]. Furthermore, DTCWT produces superior singularities of line and curve representation. Thus, discriminative feature can be extracted comparatively, which is crucial for any pattern classification problem.

Misclassification rates and higher dimensionality of features present problems concerning pattern classification. For smooth classification, dimensionality reduction techniques are employed to transform data from higher to lower dimensional spaces. PCA is the most frequently applied linear transformation and addresses these concerns. Extracted features are analyzed using PCA for feature reduction. For each MRI image from the OASIS and ADNI datasets, there are 49,152 (1536 × 32) features. After applying PCA, this is reduced to 95 × 94 for OASIS data and 172 × 171 for ADNI data.

After PCA, the classification may still not be sufficient, as PCA does not account for variability of features within a class or between classes. To ensure that the PCs are more separable, it is needed to transform data onto another space combining directions that will find axes, which will maximize the gap between different classes. Thus, LDA is applied to project PCs onto new projection axes for more effective disease classification.

TSVM is an emerging efficient pattern classification and regression algorithm in machine learning. Numerous studies have shown that TSVM is highly effective in terms of classification, regression performance, and time complexity [19, 21–23]. Hence, we have applied TSVM using linear discriminant DTCWT principal components as input features.

All programs are executed in MATLAB 2015b installed on an Intel (R) Core (TM) i3-4160 CPU system. The time complexity of the extraction of DTCWT and DWT coefficients from a 2D MRI image slice are 0.5148 and 0.5109, respectively. There is no significant difference in CPU-elapsed time when comparing transform methods. As a dimensionality reduction technique, we used PCA to omit higher dimensional input features.

In addition, it is not feasible to train and test a classifier with higher dimensional features due to elapsed time. The CPU-elapsed time to achieve TSVM classification performance was approximately 88.40 seconds without reducing dimensions. The time required for our proposed method is approximately 15.74 seconds—faster than the methods that do not employ fisher discriminant analysis.

### 3.2. Performance Evaluation

The performance of a binary classifier can be visualized using a confusion matrix, as shown in Table 4. The number of examples correctly predicted by the classifier is located on the diagonal. These may be divided into true positives (TP), representing correctly identified patients, and true negatives (TN), representing correctly identified controls. The number of examples wrongly stratified by the classifier may be divided into false positives (FP), representing controls incorrectly classified as patients, and false negatives (FN), representing patients incorrectly classified as controls.

Accuracy is determined measuring the proportion of examples that are correctly labeled by a classifier:

(13) $$\begin{array}{c} Accuracy=\frac{TP+TN}{TP+TN+FP+FN}. \end{array}$$

This may not be an ideal performance metric if the class distribution of the dataset is unbalanced.

For example, if class *C*1 is much larger than *C*2, a high accuracy value could be obtained by a classifier that labels all examples as belonging to class *C*1. Sensitivity is the rate of true positives (TP), and specificity is the rate of true negatives (TN). Sensitivity and specificity are defined as

(14) $$\begin{array}{c} sensitivity=\frac{TP}{TP+FN},\\ specificity=\frac{TN}{TN+FP}. \end{array}$$

Sensitivity measures the proportion of correctly identified patients, and specificity measures the proportion of correctly identified controls. Additionally, some other frequently used statistical performance evaluation measures such as precision, recall, f_measure, and gmean are also calculated.

These measures are defined as

(15) $$\begin{array}{c} Recall=sensitivity,\\ Precision=\frac{TP}{TP+FP},\\ f\_measure=2^{*}\left(\frac{precision^{*}recall}{precision+recall}\right),\\ gmean=sqrt\left(TP\;rate^{*}TN\;rate\right). \end{array}$$

The previous measures are likely to provide an efficient overall performance assessment of a classifier.

### 3.3. Performance of Classification

**Figure 5:**
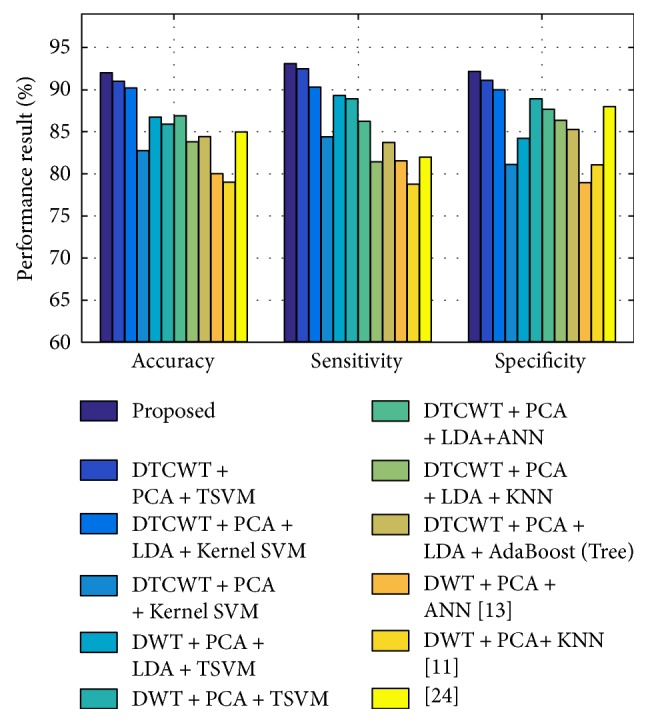
Bar chart of DTCWT-based classification performance of AD from HC over ADNI dataset.

**Figure 6:**
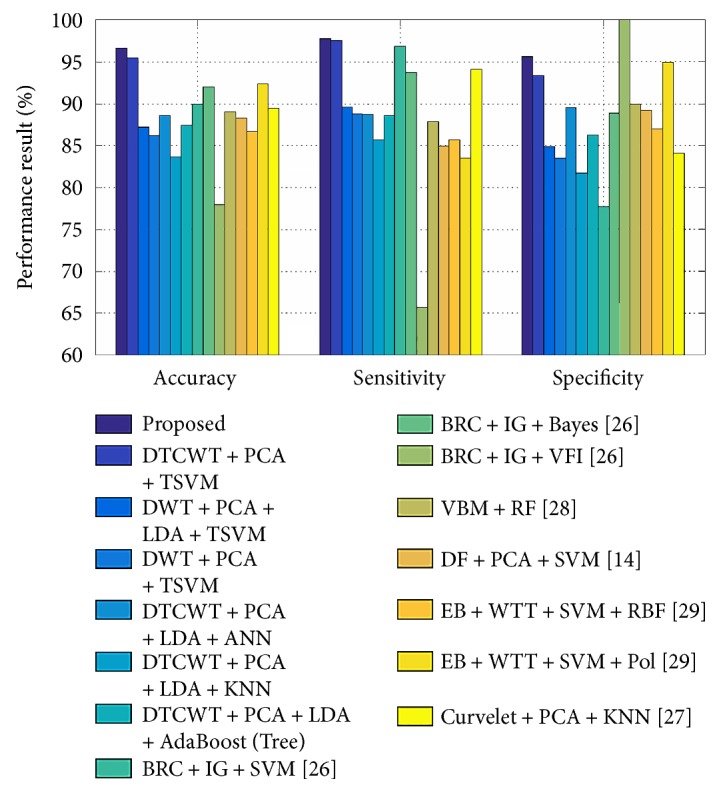
Bar chart of DTCWT-based classification performance of AD from HC over OASIS dataset.

**Figure 7:**
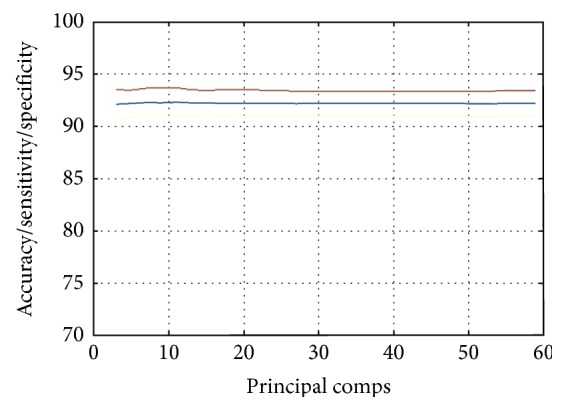
The number of principal components versus classification performance graph of proposed method.

In this study, the proposed hybrid method has been used for OASIS and ADNI data to distinguish control subjects from AD subjects. The recorded classification performance regarding accuracy (acc), sensitivity (sens), and specificity (spec) has been shown in a bar diagram in Figure 5 and in Figure 6. Performance varies depending on the principal components used for training and testing, as shown in Figure 7 for ADNI data. After testing with different PC values for both datasets, it was concluded that optimal classification performance was achieved with PC = 20. To run a strict statistical analysis, stratified cross validation (SCV) is applied. We have applied 5-fold CV to OASIS data and 10-fold CV to ADNI data, as the number of subjects in the OASIS dataset is lower than that of the ADNI dataset. 5-fold CV divides the dataset into five folds, whereas the 10-fold CV divides the dataset into ten folds.

The accuracies, sensitivities, specificities, and other statistical performance measures obtained with 10–20 runs of 10-fold SCV and 5-fold SCV are shown in Tables 5 and 6, respectively.

Although comparison with conventional methods can be difficult, we have compared our approach with some recent conventional disease detection algorithms using both datasets.

To analyze the performance over the ADNI dataset, the classification performance has been documented with both run-wise fold-wise classification, as shown in Tables 7 and 8.  Table 8 shows the classification performance where linear discriminant analysis is not used. Individual columns and rows represent the classification accuracy of the corresponding runs and folds. Consequently, accuracy is calculated taking the average of all folds and runs. The classification performance in all 10 or 5 folds of each run can be analyzed with that.

We have compared several recently used sets of algorithms and methods [11, 13, 24], using the same datasets as in this article. We have obtained a 92.65 ± 1.18% accuracy, which outperforms the DWT-based method proposed by El-Dahshan et al. [11] and Zhang et al., [13] as shown in Table 9 and Figure 5. The proposed method was also executed applying conventional DWT principal coefficients. We have seen that the DTCWT-based method outperforms DWT-based method. In addition, performance is documented without using LDA for both types of feature. However, classification performance has become more efficient when LDA-projected features are considered, as shown in Tables 5 and 9 and Figure 5. Our method has been distinguished from the volumetric feature-based research study proposed by Schmitter et al. [24], and it outperforms the results thereof, as shown in Figure 5. Additionally, our results were compared with kernel SVM-based classification and produced superior performance.

Likewise, to analyze and stratify OASIS dataset, identical methods have been used, namely run-wise and fold-wise classifications, as depicted in Tables 10 and 11.

We observed, as shown in Tables 6 and 12 and Figure 6, that our method yielded an accuracy of 96.68 ± 1.44, a sensitivity of 97.72 ± 2.34, and a specificity of 95.61 ± 1.67. This classification performance has also been documented without using LDA; however, results improve when LDA is applied on principal dual-tree complex wavelet transform coefficients or principal DWT coefficients and TSVM is used as a classifier. The result is efficient when DTCWT principal coefficients are used over DWT method.

To further verify the efficacy of the proposed method, we compared it with 12 state-of-the-art approaches, as shown in Table 12, which utilized different statistical settings.

The results show that US + SVD-PCA + SVM-DT [25] yielded an accuracy of 90%, a sensitivity of 94%, and a specificity of 71%; BRC + IG + SVM [26] achieved an accuracy of 90.00%, a sensitivity of 96.88%, and a specificity of 77.78%; and curvelet + PCA + KNN [27] obtained stratification an accuracy of 89.47%, a sensitivity of 94.12%, and a specificity of 84.09%. We observed that these methods have lower specificity compared to the other methods mentioned previously. In contrast, BRC + IG + Bayes [26] yielded higher specificity.

Similarly, BRC + IG + VFI [26] yielded a classification accuracy of 78%, sensitivity of 65.63%, and specificity of 100%. Although it yielded high specificity, accuracy and sensitivity yielded by this algorithm were comparatively poor.

All other methods achieved satisfying results. VBM + RF [28] obtained an accuracy of 89.0 ± 0.7%, a sensitivity of 87.9 ± 1.2%, and a specificity of 90.0 ± 1.1. These promising results were achieved largely due to voxel-based morphometry (VBM).

DF + PCA + SVM [14] yielded an accuracy of 88.27 ± 1.89%, a sensitivity of 84.93 ± 1.21%, and a specificity of 89.21 ± 1.63%. This method is based on a novel approach called displacement field (DF).

EB + WTT + SVM + RBF [29] obtained an accuracy of 86.71 ± 1.93%, a sensitivity of 85.71 ± 1.91%, and a specificity of 86.99 ± 2.30%; however, EB + WTT + SVM + Pol [29] yields better classification performance.

In addition, MGM + PEC + SVM [30], GEODAN + BD + SVM [30], and TJM + WTT + SVM [30] achieved approximately 92% accuracy with similarly high sensitivity and precision; specificity was not calculated for these methods.

Finally, taking classification performance into consideration, our approach outperforms all other methods analyzed here. We have also produced promising performance metrics for sensitivity and specificity. Hence, we submit that our results are either superior or comparable to the other compared methods.

## 4. Conclusions

Our proposed experiment uses LDA on the principal components of DTCWT coefficients and TSVM to stratify AD. Our proposed detection method for the ADNI dataset yielded an accuracy of 92.65 ± 1.18% with high sensitivity and specificity. Our proposed method also outperforms those of Zhang et al. [13] and El-Dahshan et al. [11] and the volumetric feature-based classification proposed by Schmitter et al. [24]. In addition, the classification performance of our proposed experiment for OASIS data performs better when compared with the several state-of-the-art approaches specified in this paper—yielding an accuracy of 96.68 ± 1.44 with similarly high sensitivity and specificity.

In the future, we will carry forward our research focusing on the following: (i) 3D DTCWT-based feature extraction with multiresolution analysis and classification and (ii) convolutional neural network- (CNN-) based classification using 3D MRI.

## Figures and Tables

**Table 1:** Summary of subject's demographics status.

	AD	Normal
Number of subjects	86	86
	43 males	46 males
	43 females	40 females
Average age	77.30	76.05
Average education points	14.65	15.93
MMSE	23.48	29.08

**Table 2:** Statistical OASIS data details used in our learning.

Factors	Normal	Very mild & mild AD
Number of patients	44	51
Age	84.40 (76–96)	82.11 (76–96)
Education	3.34 (1–5)	3.13 (1–5)
Socioeconomic status	2.31 (1–5)	2.82 (1–5)
CDR (0.5/1)	0	35/16
MMSE	28.72 (25–30)	24.82 (18–30)

**Table 3:** Clinical dementia scale.

CDR	Rank
0.5	Very mild dementia
1	Mild
2	Moderate
3	Severe

**Table 4:** Confusion matrix for a binary classifier to distinguish between two classes (S_1_ and S_2_).

True class	Predicted class	
	S_1_ (patients)	S_2_ (controls)
S_1_ (patients)	TP	FN
S_2_ (controls)	FP	TN

**Table 5:** Performance evaluation over ADNI dataset.

Methods	Accuracy	Sensitivity	Specificity	Precision	Recall	f_measure	gmean
Proposed	92.65 ± 1.18	93.11 ± 1.29	92.19 ± 1.56	92.78 ± 1.27	93.11 ± 1.29	92.63 ± 1.19	92.46 ± 1.24
DTCWT+PCA+TSVM	91.77 ± 0.85	92.48 ± 0.89	91.13 ± 1.31	91.73 ± 0.95	92.48 ± 0.89	91.72 ± 0.77	91.57 ± 0.91

**Table 6:** Performance evaluation over OASIS dataset.

Methods	Accuracy	Sensitivity	Specificity	Precision	Recall	f_measure	gmean
Proposed	96.68 ± 1.44	97.72 ± 2.34	95.61 ± 1.67	96.13 ± 1.57	97.72 ± 2.34	96.76 ± 1.51	96.56 ± 1.44
DTCWT+PCA+TSVM	95.46 ± 1.35	97.55 ± 1.26	93.36 ± 2.39	94.14 ± 2.01	97.55 ± 1.26	95.61 ± 1.28	95.29 ± 1.42

**Table 7:** Run- and fold-wise classification performance of proposed approach over ADNI dataset.

Folds	Runs	Run 1	Run 2	Run 3	Run 4	Run 5	Run 6	Run 7	Run 8	Run 9	Run 10	
Fold 1		94.44	100	100	100	94.44	88.8889	100	87.5	94.44	100	*Average accuracy* 92.659
Fold 2		100	94.117	100	88.23	82.35	94.11	81.25	94.11	94.44	88.88	
Fold 3		94.117	94.117	94.11	88.88	100	82.35	100	100	88.88	88.23	
Fold 4		94.117	88.235	88.88	94.11	100	93.75	94.117	94.11	100	82.35	
Fold 5		87.5	88.888	88.88	94.11	88.23	100	93.75	100	87.5	94.44	
Fold 6		100	94.117	87.5	88.88	100	76.47	88.23	77.77	94.11	94.44	
Fold 7		87.5	94.117	87.5	93.75	100	83.33	100	94.11	82.35	93.75	
Fold 8		87.5	100	100	88.88	100	94.44	100	83.33	87.5	94.11	
Fold 9		94.444	100	94.44	94.11	88.88	94.11	100	88.235	88.888	87.5	
Fold 10		94.444	83.333	83.33	94.11	82.35	100	88.88	94.117	100	100	
Fold-wise accuracy		93.406	93.692	92.46	92.512	93.62	90.747	94.624	91.3317	91.813	92.37	

**Table 8:** Run- and fold-wise classification performance of the DTCWT + PCA + TSVM method over ADNI dataset.

Folds	Runs	Run 1	Run 2	Run 3	Run 4	Run 5	Run 6	Run 7	Run 8	Run 9	Run 10	
Fold 1		88.88	94.11	94.11	87.5	87.5	77.77	75	100	82.35	88.88	*Average accuracy* 91.77
Fold 2		94.11	100	100	94.44	100	94.44	88.88	94.11	88.23	87.5	
Fold 3		94.11	87.5	88.23	93.75	94.11	94.11	88.88	88.23	93.75	76.47	
Fold 4		93.75	82.35	88.23	88.23	100	94.11	94.11	88.23	76.47	100	
Fold 5		88.88	94.11	94.11	83.33	82.35	94.11	82.35	88.88	94.44	100	
Fold 6		94.11	82.35	94.44	100	100	100	87.5	94.11	88.88	88.88	
Fold 7		83.33	94.44	100	100	83.33	87.5	100	88.23	100	100	
Fold 8		87.5	94.44	83.33	82.35	88.23	93.75	94.44	88.23	93.75	83.33	
Fold 9		94.44	100	94.44	88.88	100	88.23	100	82.35	88.88	100	
Fold 10		94.11	94.11	88.23	88.23	94.11	100	100	100	100	100	
Fold-wise accuracy		91.32	92.34	92.51	90.67	92.96	92.40	91.11	91.24	90.67	92.50	

**Table 9:** Classification performance of AD from HC over ADNI data.

Methods	Accuracy	Sensitivity	Specificity
Proposed	92.65 ± 1.18	93.11 ± 1.29	92.19 ± 1.56
DTCWT + PCA + TSVM	91.77 ± 0.85	92.48 ± 0.89	91.13 ± 1.31
DTCWT + PCA + LDA + Kernel SVM	90.181 ± 0.97	90.276 ± 1.60	90.101 ± 1.23
DTCWT + PCA + Kernel SVM	82.74 ± 1.24	84.43 ± 1.51	81.18 ± 1.85
DWT + PCA + LDA + TSVM	86.75 ± 1.69	89.32 ± 1.43	84.23 ± 2.21
DWT + PCA + TSVM	85.88 ± 1.16	88.93 ± 1.61	88.93 ± 2.02
DTCWT + PCA + LDA + ANN	86.97 ± 1.30	86.25 ± 1.78	87.72 ± 3.51
DTCWT + PCA + LDA + KNN	83.89 ± 0.75	81.41 ± 1.33	86.34 ± 1.08
DTCWT + PCA + LDA + AdaBoost (tree)	84.48	83.72	85.26
DWT + PCA + ANN [13]	80.05 ± 0.72	81.538 ± 1.41	78.974 ± 1.09
DWT + PCA + KNN [11]	79.964 ± 1.19	78.771 ± 2.37	81.08 ± 1.67
[24]	85	82	88

**Table 10:** Run- and fold-wise classification performance of the proposed approach over OASIS dataset.

Folds	Runs	Run 1	Run 2	Run 3	Run 4	Run 5	Run 6	Run 7	Run 8	Run 9	Run 10	
Fold 1		94.44	94.44	100	88.23	100	100	100	94.11	100	100	*Average accuracy* 96.58
Fold 2		94.11	100	88.23	88.88	88.88	100	94.11	100	88.88	93.75	
Fold 3		94.44	94.11	100	94.11	100	94.44	100	94.11	100	100	
Fold 4		100	100	100	94.44	100	94.44	100	100	100	100	
Fold 5		100	88.88	100	100	94.44	100	94.11	100	94.11	94.44	
Fold-wise accuracy		96.60	95.49	97.64	93.13	96.66	97.77	97.64	97.64	96.60	97.63	

**Table 11:** Run- and fold-wise classification performance of the DTCWT + PCA + TSVM method over OASIS dataset.

Folds	Runs	Run 1	Run 2	Run 3	Run 4	Run 5	Run 6	Run 7	Run 8	Run 9	Run 10	
Fold 1		100	94.11	94.44	100	94.44	94.44	94.11	94.44	88.8	94.44	*Average accuracy* 95.46
Fold 2		100	100	88.23	94.44	94.44	100	94.11	94.44	100	94.44	
Fold 3		94.44	83.33	94.44	94.11	100	94.44	94.44	94.11	94.44	100	
Fold 4		94.44	100	94.44	94.11	94.44	88.88	100	100	100	88.88	
Fold 5		94.11	100	94.11	94.44	100	87.5	100	94.44	100	100	
Fold-wise accuracy		96.60	95.49	93.13	95.42	96.66	93.05	96.53	95.49	96.66	95.55	

**Table 12:** Algorithm performance comparison over OASIS MRI data.

Algorithm	Accuracy	Sensitivity	Specificity	Precision
Proposed	**96.68 ± 1.44**	**97.72 ± 2.34**	**95.61 ± 1.67**	**96.13 ± 1.57**
DTCWT + PCA + TSVM	95.46 ± 1.35	97.55 ± 1.26	93.36 ± 2.39	94.15 ± 2.01
DWT + PCA + LDA + TSVM	87.23 ± 1.65	89.61 ± 2.25	84.85 ± 1.66	86.66 ± 1.99
DWT + PCA + TSVM	86.19 ± 1.50	88.83 ± 1.98	83.5 ± 1.87	85.66 ± 1.84
DTCWT + PCA + LDA + ANN	88.59 + 2.08	88.75 + 2.75	89.55 + 3.96	NA
DTCWT + PCA + LDA + KNN	83.69 + 1.57	85.7 + 1.94	81.8 + 1.45	NA
DTCWT + PCA + LDA + AdaBoost (tree)	87.45	88.59	86.26	NA
BRC + IG + SVM [26]	90.00 (77.41, 96.26)	96.88 (82.01, 99.84)	77.78 (51.92, 92.63)	NA
BRC + IG + Bayes [26]	92.00 (79.89, 97.41)	93.75 (77.78, 98.27)	88.89 (63.93, 98.05)	NA
BRC + IG + VFI [26]	78.00 (63.67, 88.01)	65.63 (46.78, 80.83)	100.00 (78.12, 100)	NA
MGM + PEC + SVM [30]	92.07 ± 1.12	86.67 ± 4.71	N/A	95.83 ± 5.89
GEODAN + BD + SVM [30]	92.09 ± 2.60	80.00 ± 4.00	NA	88.09 ± 5.33
TJM + WTT + SVM [30]	92.83 ± 0.91	86.33 ± 3.73	N/A	85.62 ± 0.85
VBM + RF [28]	89.0 ± 0.7	87.9 ± 1.2	90.0 ± 1.1	NA
DF + PCA + SVM [14]	88.27 ± 1.9	84.93 ± 1.21	89.21 ± 1.6	69.30 ± 1.91
EB + WTT + SVM + RBF [29]	86.71 ± 1.93	85.71 ± 1.91	86.99 ± 2.30	66.12 ± 4.16
EB + WTT + SVM + Pol [29]	92.36 ± 0.94	83.48 ± 3.27	94.90 ± 1.09	82.28 ± 2.78
Curvelet + PCA + KNN [27]	89.47	94.12	84.09	NA
US + SVDPCA + SVM-DT [25]	90	94	71	NA

## Additional Points

*Data Access*. Data used in reparation of this article were obtained from the Alzheimer's Disease Neuroimaging Initiative (ADNI) database (http://adni.loni.usc.edu). A complete listing of ADNI investigators can be found at https://adni.loni.usc.edu/wp-content/uploads/how_to_apply/ADNI_Acknowledgement_List.pdf.
